# Introductory lecture: Diagnosis of food allergy: when is an oral challenge positive

**DOI:** 10.1186/2045-7022-1-S1-S43

**Published:** 2011-08-12

**Authors:** Bodo Niggemann

**Affiliations:** 1 Pädiatrische Allergologie und Pneumologie, Hedwig-von-Rittberg-Zentrum, DRK-Kliniken Berlin, Berlin, Germany

## 

Oral food challenges still remain the gold standard in the diagnosis of food related symptoms and are performed to obtain a clear “yes or no” response. However, this is often difficult to achieve, and so proposals may be appropriate for criteria on when to stop oral food challenges and declare a challenge as positive or negative. In daily practice it makes sense to challenge until clear objective symptoms occur without harming the patient. Clinical symptoms should be objective and/or: (a) severe or (b) reproducible or (c) persisting. A sensitive parameter for a beginning clinical reaction is a general change of mood. The sooner symptoms appear, the more likely they are to represent a “true” positive reaction, and the more organ systems are involved the easier it is to assess an oral food challenge as positive. In the case of subjective symptoms, the number of placebo doses should be increased. In unclear situations, the observation time until the next dose should be prolonged or the same dose repeated. Transient objective clinical symptoms usually end up in a positive challenge result. There are a number of causes for false positive and false negative challenge results, which should be considered. The aim of all oral challenge testing should be to hold the balance between two conflicting aspects: on the one hand the need to achieve clear and justified results from oral food challenges in order to avoid unnecessary diets, and on the other hand to protect patients from any harm caused by high doses of a potentially dangerous food.

